# Sentinel Lymph Node Biopsy Is Feasible in Cervical Cancer Laparoscopic Surgery: A Single-Center Retrospective Cohort Study

**DOI:** 10.1155/2021/5510623

**Published:** 2021-04-16

**Authors:** Hongyi Hou, Yibo Dai, Sichen Liang, Zhiqi Wang, Jianliu Wang

**Affiliations:** Department of Obstetrics and Gynecology, Peking University People's Hospital, Beijing, China

## Abstract

**Methods:**

A total of 100 cervical cancer patients undergoing laparoscopic surgery with SLN biopsy were included. Indocyanine green, carbon nanoparticles (CNPs), and a combination of both were used during surgeries. Detection rates, sensitivity, negative predictive value (NPV) of SLN biopsy, and related factors were analyzed.

**Results:**

The overall and bilateral SLN detection rates were 92% (92/100) and 74% (74/100), respectively. Combined tracers had higher bilateral SLN detection rates than CNPs alone (*p*=0.005). Menopause and lymph node metastasis were associated with lower overall and bilateral SLN detection rates (*p* < 0.05). SLN biopsy sensitivity and NPV for lymph node metastasis in patients with at least one detected SLN were 81.8% (9/11) and 97.3% (72/74), respectively. Among those with bilateral detected SLNs, higher sensitivity and NPV of 87.5% (7/8) and 98.3% (57/58) were observed, respectively. SLN algorithm can ensure that all patients with lymph node metastasis are detected by SLN biopsy.

**Conclusion:**

SLN biopsy appears to be safe and effective for specific cervical cancer patients with high detection rates and NPV in laparoscopic surgery, especially for those with detected bilateral SLNs and undergoing the SLN algorithm. Selecting suitable patients for SLN mapping has prospects for clinical application.

## 1. Introduction

Cervical cancer is one of the most common gynecologic malignancies around the world [1, 2]. Since lymph node metastasis is one of the most important prognostic predictors of early-stage cervical cancer, it is closely associated with adjuvant therapy strategy [3, 4], thus making lymph node resection the most important step in comprehensive surgical staging of cervical cancer. Nevertheless, lymph node positivity rate is relatively low in early-stage cervical cancer patients. In addition, systemic lymph node resection is associated with longer surgical time, higher bleeding volume, higher rates of intraoperative subsidiary injures, and destruction of normal barrier functions in the lymph nodes [5].

SLN biopsy has gained popularity in recent years. Since first introduced in the 1960s, studies on SLN have been performed in breast cancer, melanoma, and vulvar cancer, and standards for SLN biopsy have been set up in clinical practice. SLN biopsy is now recommended by the National Comprehensive Cancer Network (NCCN) guidelines for stage I cervical cancer patients (category 2A) [6]. However, there is still no consensus on the standard SLN biopsy method in cervical cancer, and differences exist among current study results.

Based on retrospective data from Peking University People's Hospital, the present study compared different tracers and analyzed clinicopathological factors associated with SLN detection rates, sensitivity, and NPV for SLN biopsy in cervical cancer laparoscopic surgery.

## 2. Materials and Methods

### 2.1. Patient Selection and Data Collection

One hundred pathologically confirmed cervical cancer patients who underwent SLN mapping during laparoscopic surgeries in Peking University People's Hospital from July 2015 to June 2018 were retrospectively reviewed. Clinicopathological information was collected, including age, body mass index, gravidity, parity, medical history, history of cervical conization, neoadjuvant chemotherapy, preoperative radiotherapy, radiography, surgeries, SLN tracers, numbers of resected lymph nodes, stage, tumor size, histology, grade, lymphovascular space invasion, depth of cervical stromal invasion, and lymph node metastasis. The study was approved by the Institutional Review Board of Peking University People's Hospital (approval number: 2018PHD003-01).

### 2.2. Reagents and Instruments

CNPs injection (50 mg/1 ml per piece) was from Chongqing Lummy Pharmaceutical Co., Ltd. (approval number: H20041829). Indocyanine green (ICG) injection (25 mg per piece) was from (Liaoning) Pharmaceutical Co., Ltd. and was dissolved in 10 ml 0.9% NaCl solution before use. The intraoperative fluorescence imaging system (PC9000) was from Novadaq Technologies Inc.

### 2.3. Surgical Procedures

All 100 eligible patients received laparoscopic surgery. After successful anesthesia, SLN tracers were injected into the cervix. In the single-tracer method, 0.2 ml CNPs or ICG solution was injected into 3, 6, 9, and 12 o'clock of the cervix with a depth of 2-3 mm, respectively (0.8 ml totally). In the combination-tracing method, CNPs were injected into 3, 6, 9, and 12 o'clock firstly, followed by the injection of ICG solution to 2, 4, 8, and 10 o'clock of the cervix. Tracers were injected near the tumor to avoid being injected into the tumor tissue. After injection, the cervix was compressed for 30 seconds.

**Figure 1:**
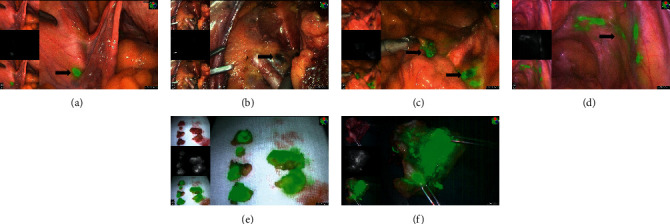
The mapped sentinel lymph nodes, lymph vessels, and uterus. (a) The sentinel lymph nodes mapped by ICG. (b) The sentinel lymph nodes mapped by CNPs. (c) The sentinel lymph nodes mapped by ICG and CNPs. (d) The lymph vessels mapped by ICG. (e) The resected sentinel lymph nodes detected by ICG. (f) The removed uterus injected by ICG.

During the surgery, the retroperitoneum was dissected, and SLNs were searched along the lymphatic drainage pathway. The anatomical sites, numbers of SLNs, and time to detection were recorded. According to the NCCN guidelines' SLN algorithm [6], all detectable SLNs and enlarged or suspicious nodes regardless of mapping were removed; if no SLN was detected in either side of the pelvis, a side-specific systemic lymph node resection was conducted; the tumor and parametrium were resected completely. The mapped SLNs, lymph vessels, and uterine are shown in Figure 1.

For stage IA1 patients with no lymphovascular space invasion in the preoperative biopsy, extrafascial hysterectomy and SLN biopsy were conducted. For stage IA1 patients with lymphovascular space invasion or higher stages, radical hysterectomy and systemic pelvic lymph node dissection were performed after SLN resection, and para-aortic lymph node biopsy was conducted when necessary. Systemically resected pelvic lymph nodes included external iliac, internal iliac, obturator fossa, deep inguinal, and common iliac lymph nodes.

All surgeries were performed by three experienced gynecologic oncologists in our center.

### 2.4. Pathological Review

Routine hemotoxin and eosin staining was used. We did not conduct the SLN ultrastaging and immunohistochemistry staining in this study. All pathological reviews were finished by two independent gynecologic pathologists in the Department of Pathology of Peking University People's Hospital. When discordance occurred, a third senior pathologist was invited to decide the diagnosis.

### 2.5. Evaluation of SLN Biopsy

Overall SLN detection rate refers to the percentage of patients with successfully detected SLNs in all eligible patients. Bilateral SLN detection rate refers to the percentage of patients with successfully detected SLNs on both sides of pelvis in all eligible patients. Sensitivity refers to the percentage of patients with positive SLNs in routine pathological examination among those with positive pelvic lymph nodes. NPV refers to the percentage of patients with negative pelvic lymph nodes among those whose SLNs were negative in routine pathological examination.

### 2.6. Statistical Methods

The *χ*^2^ test (with continuity adjustment) and Fisher's exact test were used for comparing categorical variables, and partition of *χ*^2^ test (Bonferroni method) was used for intergroup comparisons. Student's *t*-test was used for comparing continuous variables. Multivariable logistic regression was conducted to analyze independent predictors of SLN detection rates. All statistical analyses were performed using Statistical Package for the Social Sciences (SPSS) Software 23.0 (IBM Corporation, Armonk, NY). All *p* values were two-sided, and *p* < 0.05 was considered statistically significant.

## 3. Results

**Figure 2:**
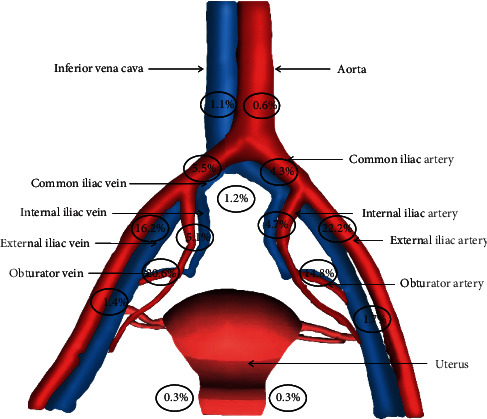
The distribution of sentinel lymph nodes.

The clinicopathologic characteristics of 100 eligible patients are listed in Table 1. There were no previous histories of malignant tumors, pelvic lymphadenectomy, inguinal lymphadenectomy, or other surgeries that might change uterine lymphatic drainage in the 100 patients. The staging of all cases was based on the 2018 International Federation of Gynecology and Obstetrics (FIGO) staging system [7]. 2,875 pelvic lymph nodes were removed during surgeries, and the median number of pelvic lymph nodes removed was 29 (range: 24–35) in each patient. The median surgical time to complete the SLN mapping was 30 min (range: 25–40 min). A total of 722 sentinel lymph nodes were removed, with a median number of 7 (range: 4–11) for each patient. The most common location of SLNs was the external iliac region (277/722, 38.4%), followed by obturator fossa (256/722, 35.5%), common iliac (71/722, 9.8%), internal iliac (71/722, 9.8%), deep inguinal (22/722, 3.0%), presacral (9/722, 1.2%), inferior vena cava (8/722, 1.1%), para-aortic (4/722, 0.6%), and parametrial (4/722, 0.6%) regions. The distribution of SLNs is presented in Figure 2.

The overall detection rate of SLNs was 92% (92/100). SLNs were found on both sides of the pelvis in 74 patients (74%). There were 85 patients undergoing combined tracers of ICG and CNPs, 8 patients with ICG tracer alone, and 7 patients undergoing CNPs tracer alone. The SLN detection rates according to the different tracer methods are listed in Table 2. The percentage of patients with at least one detected SLN did not differ significantly among the three groups (*p*=0.262). However, the difference of bilateral detection rates among the three groups was statistically significant (*p*=0.014). Bonferroni-corrected threshold *α* = 0.0167 was applied in analyses stratified according to different tracers. The bilateral detection rate was significantly higher by combined tracers compared with CNPs tracer alone (75.3% versus 54.3%, *p*=0.005).

Clinicopathological factors influencing the detection rates of SLNs were analyzed (Table S1). Factors with *p* < 0.1 in univariate analyses or those with potential influence according to our clinical experience were included in the logistic regression model. The results suggested that menopause (OR = 0.02, 95% CI 0.001–0.47, *p*=0.016; OR = 0.30, 95% CI 0.09–0.93, *p*=0.038) and lymph node metastasis (OR = 0.03, 95% CI 0.002–0.71, *p*=0.030; OR = 0.17, 95% CI 0.03–0.92, *p*=0.040) were associated with lower overall and bilateral SLN detection rates (Table 3). The overall SLN detection rate was 98.4% in premenopausal patients and 82.1% in postmenopausal patients, and the bilateral SLN detection rates were 82.0% and 61.5%, respectively. The patients with positive pelvic lymph nodes had lower overall and bilateral SLN detection rates compared with those without positive lymph nodes (73.3% versus 95.3%; 53.3% versus 77.6%).

**Figure 3:**
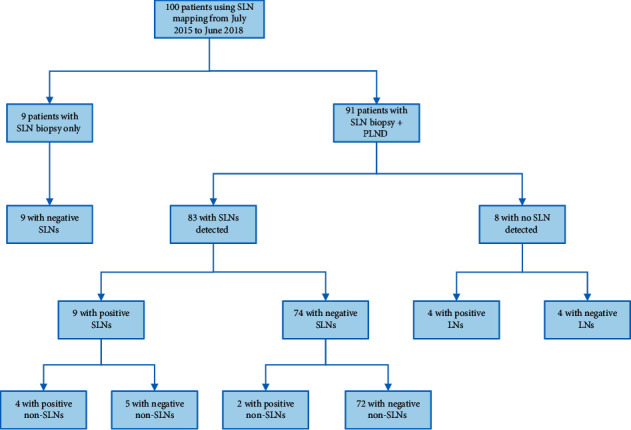
The results of SLN biopsy and pelvic lymphadenectomy. SLN, sentinel lymph node; PLND, pelvic lymph node dissection; LN, lymph node.

The results of SLN biopsy and pelvic lymphadenectomy are shown in Figure 3. Among the 83 patients with at least one SLN detected undergoing SLN mapping followed by systemic pelvic lymphadenectomy, 9 had positive SLNs, and 2 cases were false negative. Therefore, the sensitivity and NPV of SLN biopsy were 81.8% (9/11) and 97.3% (72/74), respectively. Among the 65 patients with bilateral SLNs detected, one had a false-negative result, yielding a sensitivity of 87.5% (7/8) and an NPV of 98.3% (57/58). Diagnostic accuracy of SLN biopsy is listed in Table 4.

Two patients showed false-negative results. A total of 8 negative SLNs were detected in patient A, located in the left external iliac, right external iliac, right internal iliac, and right obturator region. But a single positive non-SLN was found in right parametrial area. Patient B had 3 negative SLNs detected in the right external iliac and right para-aortic region, but 5 positive non-SLNs were found in the left hemi-pelvis (left obturator region). If the NCCN guidelines' SLN algorithm had been applied in this cohort, the false-negative patient seems to be 0 and the sensitivity and NPV are 100%.

**Figure 4:**
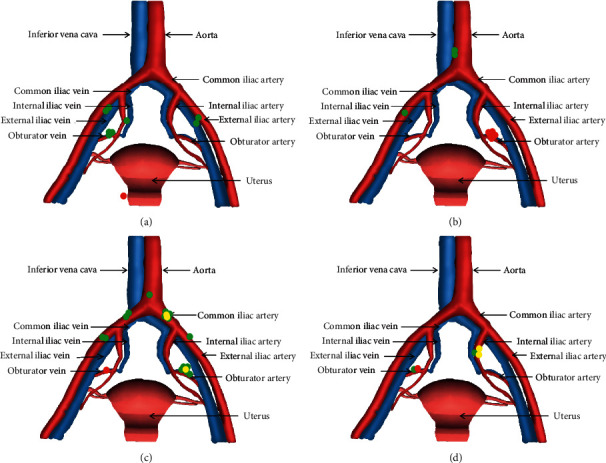
The patients with false-negative sentinel lymph nodes, 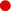 positive non-sentinel lymph nodes, 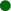 negative sentinel lymph nodes and 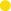 positive sentinel lymph nodes. (a) Patient A. (b) Patient B. (c) Patient C. (d) Patient D.

SLNs were detected in 148 hemi-pelvises. 13 hemi-pelvises had positive lymph nodes, 10 of which had positive SLNs. Side-specific sensitivity was 76.9% (10/13), and NPV was 97.8% (135/138). The 3 false-negative hemi-pelvises were from 3 different patients. Patient C had one positive non-SLN located in the right obturator region, 4 negative SLNs located in the right common iliac and right external iliac area, and 3 positive SLNs located in the left common iliac and left obturator region. There was one positive non-SLN in the right obturator region of patient D who had 2 negative SLNs in the right obturator and left internal iliac region and 2 positive SLNs in the left internal iliac region. The third patient was the above patient A. Lymph node statuses of the four patients are shown in Figure 4.

## 4. Discussion

This study aimed to explore the detection rates, diagnostic accuracy, and the associated factors of SLN biopsy in order to analyze its feasibility and clinical value in cervical cancer. The overall and bilateral SLN detection rates of 92% and 75% were observed, which were generally consistent with the results of previous studies [8–10]. Combined technique could bring a higher bilateral detection rate than CNPs alone but not ICG alone. Patients after their menopause or with metastatic pelvic lymph nodes presented the significantly lower overall and bilateral SLN detection rates. When SLNs were detected on bilateral pelvis and SLN mapping algorithm was adhered to, SLN biopsy was more reliable.

Near-infrared fluorescence imaging has the potential for the identification of anatomical structures covered under a layer of fatty tissue, with a maximum penetration depth of 10 mm [11]. Previous studies emphasized the advantages of ICG over other tracers for better SLN detection in cervical and endometrial cancer, with the reported detection rates ranging from 78% to 86% [12–14]. Imboden et al. [15] used Tc-99 radiotracer combined with blue dye for SLN mapping in a group of 36 cervical cancer patients and ICG alone in another group of 22 patients. The overall detection rate for dual injection was 83%, while for ICG alone it was 95.5%. The bilateral detection rate was 61% and 95.5%, respectively (*p* < 0.05). In a meta-analysis of 6 studies, including 538 patients with cervical and endometrial cancers, the overall and bilateral SLN detection rates were both significantly higher in the ICG group than those in the blue dye group (*p* < 0.001). Meanwhile the advantage was not shown when compared with Tc-99 alone or combined use of blue dye and Tc-99 (*p* > 0.05) [16].

We found that menopausal state was associated with lower SLN detection rates, which was, to some extent, in accordance with previous findings [8, 17]. Possible reasons include weakened lymphatic drainage in atrophy of the cervix with the decline of estrogen level after menopause, which might affect the tracers' spread. Lymph node metastasis was also proved to influence the SLN detection by previous studies [18]. In the study by Malur et al. [19], the SLNs in 50 patients with cervical cancer were mapped with Tc-99 and/or blue dye. The overall SLN detection rate was 78% (39/50). Among the 10 patients with metastatic lymph nodes, 4 patients had no SLNs detected. Tumor thrombus will obstruct and redistribute lymphatic drainage so that tracers fail to reach SLNs normally along the lymphatic vessels, leading to the failure of SLN mapping. In breast cancer and penile cancer, this hypothesis was generally accepted, and SLN biopsy was not applicable if there were suspected metastatic lymph nodes in preoperative assessment [20–22]. So it is imperative to identify the patients with high risk of lymph node metastasis before cervical cancer surgery and assess the feasibility of SLN biopsy.

Diagnostic accuracy of SLN biopsy procedure is critical and should be as high as possible. To reduce the false-negative rate, detecting SLNs on both sides of the pelvis is an essential factor. The study by Lecuru and colleagues supported this view [8]. Among 139 patients of early-stage cervical cancer, 2 cases were false negative, generating a sensitivity of 92% and an NPV of 98.2%. However, among the 104 patients with SLNs detected bilaterally, none had a false-negative result. In a study conducted by AGO, the sensitivity of SLN biopsy increased from 69.6% to 87.2% in patients with bilaterally detected SLNs, compared with those whose SLNs were mapped in one side of the pelvis (*p*=0.046). An increase in NPV from 91.0% to 96.5% (*p*=0.062) was also observed [23]. Our results further supported the above findings. Besides, in previous studies, SLN ultrastaging and molecular detection methods also increased the diagnostic accuracy of SLN biopsy [23, 24]. Nevertheless, the effects of micrometastasis and isolated tumor cells of lymph nodes on cervical cancer prognosis were still debatable.

NCCN guidelines' SLN mapping algorithm is fundamental for SLN biopsy to replace systemic lymphadenectomy in patients with early-stage cervical cancer [6]. Cormier's [9] study on SLN mapping algorithm for early-stage cervical cancer showed that, among 122 patients, 3 cases were false negative. Two cases had positive non-SLNs located in the parametrial area, and the positive non-SLNs of another case were at the side without SLNs detected. If the SLN mapping algorithm was implemented, the three false-negative patients could be identified and receive necessary adjuvant treatment. According to the SLN algorithm, the two false-negative patients in our study, patient A and patient B, could be identified, so that the sensitivity and NPV were both 100%. As for patients C and D, the final lymph node status was not affected, even though they had false-negative hemi-pelvises. Both of them had neoadjuvant chemotherapy history and suspected enlarged pelvic lymph nodes shown by pelvic Magnetic Resonance Imaging before surgery. A previous study thought that neoadjuvant chemotherapy could reduce the detection of pelvic lymph node metastasis [19].

In this study, all surgeries were done by 3 experienced gynecologic oncologists from our center, which guaranteed consistency and comparability among cases. The detailed sites of SLNs were documented for all patients, which helped increase the accuracy of this study; and we found some significant clinicopathological factors associated with SLN detection rates in cervical cancer, which may help screen suitable candidates for SLN biopsy in the future. But there were still some limitations. Firstly, the cohort's size was relatively small, especially as to those treated with one single tracer or patients with specific and advanced clinicopathological features. No significant influence of these factors on SLN detection rates was observed, possibly due to the limited sample size. Secondly, all surgeries were done by the laparoscopic approach in this study. Further studies exploring the application of SLN biopsy in open surgery may be necessary. Thirdly, as a retrospective research, the study is limited in controlling confounders. But statistical methods were adopted, and the results from our work may be helpful for further prospective cohort design.

## 5. Conclusions

In conclusion, SLN biopsy is feasible in cervical cancer laparoscopic surgery. Premenopausal patients or those without lymph node metastasis have higher SLN detection rates. Detecting SLNs bilaterally and following SLN mapping algorithm make SLN biopsy more reliable. A larger prospective cohort is essential for further demonstrating these results.

## Figures and Tables

**Table 1:** Clinicopathological characteristics in 100 cervical cancer patients.

Characteristic	Value
Mean age (years)	47.2 ± 9.7

Menopause	
No	61 (61.0)
Yes	39 (39.0)

Mean body mass index (kg/m^2^)	23.9 ± 3.3

Median gravidity	3 (2.4)

Median parity	1 (1.2)

Conization history	
No	85 (85.0)
Yes	15 (15.0)

Neoadjuvant chemotherapy	
No	76 (76.0)
Yes	24 (24.0)

Preoperative radiotherapy	
No	97 (97.0)
Yes	3 (3.0)

Sentinel lymph node biopsy only	
No	91 (91.0)
Yes	9 (9.0)

Stage	
IA1	11 (11.0)
IA2	3 (3.0)
IB1	27 (27.0)
IB2	26 (26.0)
IB3	9 (9.0)
IIA1	5 (5.0)
IIA2	3 (3.0)
IIB	1 (1.0)
IIIC1	13 (13.0)
IIIC2	2 (2.0)

Tumor size (cm)	
<2	45 (45.0)
≥2∼<4	39 (39.0)
≥4	16 (16.0)

Histologic type	
Squamous carcinoma	76 (76.0)
Adenocarcinoma	14 (14.0)
Adenosquamous carcinoma	4 (4.0)
Others	6 (6.0)

Grade	
1	26 (26.0)
2	41 (41.0)
3	33 (33.0)

Lymphovascular space invasion	
No	52 (52.0)
Yes	48 (48.0)

Depth of cervical invasion	
<1/2	62 (62.0)
≥1/2	38 (38.0)

Lymph node status	
Negative	85 (85.0)
Positive	15 (15.0)

The values were presented as mean ± standard deviation, median (minimum, maximum), or number (%), unless otherwise indicated.

**Table 2:** Sentinel lymph node detection rates according to different tracer methods.

Tracer	*N*	Overall detection rate (*n*, %)	*χ* ^2^	*p* value	Bilateral detection rate (*n*, %)	*χ* ^2^	*p* value
ICG	93	82, 88.2	2.702	0.262	61, 65.6	8.537	0.014
CNPs	92	77, 83.7			50, 54.3^*∗*^		
ICG + CNPs	85	78, 91.8			64, 75.3^*∗*^		

The 93 cases of ICG included 85 with combined tracers and 8 with ICG alone. The 92 cases of CNPs included 85 with combined tracers and 7 with CNPs alone. ICG detected at least one SLN in 82 patients including 75 with combined tracers and 7 with ICG alone. ICG detected bilateral SLNs in 61 patients including 55 with combined tracers and 6 with ICG alone. CNPs detected at least one SLN in 77 patients including 70 with combined tracers and 7 with CNPs alone. CNPs detected bilateral SLNs in 50 patients including 46 with combined tracers and 4 with CNPs alone. ^*∗*^CNPs versus ICG + CNPs, *p*=0.005. SLN, sentinel lymph node; ICG, indocyanine green; CNPs, carbon nanoparticles.

**Table 3:** Logistic regression for factors affecting overall and bilateral detection rates of sentinel lymph nodes.

Characteristic	Overall detection rate		Bilateral detection rate	
	OR (95% CI)	*p* value	OR (95% CI)	*p* value
Menopause				
No	1		1	
Yes	0.02 (0.001–0.47)	0.016	0.30 (0.09–0.93)	0.038

Body mass index (kg/m^2^)				
<25.0	1		1	
≥25.0	1.00 (0.13–7.84)	0.997	2.07 (0.66–6.45)	0.212

Conization history				
No	1		1	
Yes		0.999		0.998

Tumor size (cm)		0.186		0.221
<2	1		1	
≥2∼<4	0.04 (0.001–1.28)	0.068	1.20 (0.28–5.13)	0.803
≥4	0.37 (0.01–13.14)	0.583	0.36 (0.08–1.56)	0.172

Grade		0.313		0.089
1	1		1	
2		0.998	0.17 (0.03–1.06)	0.058
3		0.998	0.49 (0.07–3.19)	0.451

Lymphovascular space invasion				
No	1		1	
Yes	9.37 (0.48–182.95)	0.140	3.07 (0.74–12.72)	0.121

Depth of cervical invasion				
<1/2	1		1	
≥1/2	0.78 (0.04–14.51)	0.865	0.73 (0.18–2.97)	0.662

Lymph node metastasis				
No	1		1	
Yes	0.03 (0.002–0.71)	0.030	0.17 (0.03–0.92)	0.040

**Table 4:** Diagnostic accuracy of lymph node metastasis using SLN biopsy.

		83 patients with at least one SLN detected							65 patients with bilateral SLNs detected				
		Pelvic lymph node		*n*	Sensitivity	NPV			Pelvic lymph node		*n*	Sensitivity	NPV
		+	−						+	−			
SLN	+	9	0	9	81.8% (9/11)	97.3% (72/74)	SLN	+	7	0	7	87.5% (7/8)	98.3% (57/58)
	−	2	72	74				−	1	57	58		
*n*		11	72	83			*n*		8	57	65		

+, positive; −, negative. SLN, sentinel lymph node; NPV, negative predictive value.

## Data Availability

The data are available upon request to the corresponding author.

## Supplementary Materials

Supplementary Materials The clinicopathological factors influencing the detection rates of SLNs were analyzed in the supplementary file.
