# Supplementary material for: Sentinel Lymph Node Biopsy Is Feasible in Cervical Cancer Laparoscopic Surgery: A Single-Center Retrospective Cohort Study
Source: J Oncol. 2021 Apr 16;2021:5510623. doi: 10.1155/2021/5510623 (PMC8064774; doi:10.1155/2021/5510623)
Supplement: Supplementary Materials — The clinicopathological factors influencing the detection rates of SLNs were analyzed in the supplementary file. [file 5510623.f1.docx]

Table S1. Univariate analysis for factors affecting overall and bilateral SLN detection rates

| Characteristic | Overall detection rate | χ^2^ | p value | Bilateral detection rate | χ^2^ | p value |
| --- | --- | --- | --- | --- | --- | --- |
| Menopause  No  Yes | 98.4% (60/61)  82.1% (32/39) | 6.525 | 0.011 | 82.0% (50/61)  61.5% (24/39) | 5.160 | 0.035 |
| Body mass index (kg/m^2^)  < 25.0  ≥ 25.0 | 93.4% (57/61)  89.7% (35/39) | - | 0.708 | 70.5% (43/61)  79.5% (31/39) | 1.001 | 0.317 |
| Surgical experience |  |  |  |  |  |  |
| The first 10 operations  The last 90 operations | 90.0% (9/10)  92.2% (83/90) | - | 0.583 | 70.0% (7/10)  74.0% (67/90) | 0.000 | 1.000 |
| The first 20 operations  The last 80 operations | 95.0% (19/20)  91.3% (73/80) | - | 1.000 | 65.0% (13/20)  76.3% (61/80) | 1.052 | 0.393 |
| The first 30 operations  The last 70 operations | 96.7% (29/30)  90.0% (63/70) | - | 0.429 | 76.7% (23/30)  72.9% (51/70) | 0.158 | 0.806 |
| The first 40 operations  The last 60 operations | 95.0% (38/40)  90.0% (54/60) | 0.277 | 0.598 | 80.0% (32/40)  70.0% (42/60) | 1.247 | 0.353 |
| The first 50 operations  The last 50 operations | 92.0% (46/50)  92.0% (46/50) | 0.000 | 1.000 | 76.0% (38/50)  72.0% (36/50) | 0.208 | 0.802 |
| Conization history  No  Yes | 90.6% (77/85)  100.0% (15/15) | - | 0.602 | 69.4% (59/85)  100.0% (15/15) | - | 0.010 |
| Neoadjuvant chemotherapy  No  Yes | 90.8% (69/76)  95.8% (23/24) | 0.131 | 0.707 | 71.1% (54/76)  83.3% (20/24) | 0.863 | 0.353 |
| Pre-operative radiotherapy  No  Yes | 91.8% (89/97)  100.0% (3/3) | - | 1.000 | 74.2% (72/97)  66.7% (2/3) | 0.000 | 1.000 |
| Tumor size (cm)  < 2  ≥ 2 ~ < 4  ≥ 4 | 97.8% (44/45)  84.6% (33/39)  93.8% (15/16) | 4.649 | 0.078 | 80.0% (36/45)  74.4% (29/39)  56.3% (9/16) | 3.465 | 0.190 |
| Histologic type  Squamous carcinoma  Adenocarcinoma  Adenosquamous carcinoma  Others | 92.1% (70/76)  92.9% (13/14)  100.0% (4/4)  83.3% (5/6) | 1.403 | 0.627 | 73.7% (56/76)  71.4% (10/14)  100.0% (4/4)  66.7% (4/6) | 1.399 | 0.808 |
| Grade  1  2  3 | 100.0% (26/26)  92.7% (38/41)  84.8% (28/33) | 4.275 | 0.108 | 92.3% (24/26)  63.4% (26/41)  72.7% (24/33) | 7.986 | 0.021 |
| Lymphovascular space invasion  No  Yes | 96.2% (50/52)  87.5% (42/48) | 1.500 | 0.221 | 75.0% (39/52)  72.9% (35/48) | 0.056 | 0.824 |
| Depth of cervical invasion  < 1/2  ≥ 1/2 | 98.4% (61/62)  81.6% (31/38) | 6.904 | 0.009 | 79.0% (49/62)  65.8% (25/38) | 2.147 | 0.164 |
| Lymph node metastasis  No  Yes | 95.3% (81/85)  73.3% (11/15) | - | 0.017 | 77.6% (66/85)  53.3% (8/15) | 3.917 | 0.060 |

The operations were sorted in chronological order.

SLN, sentinel lymph node.
